# Identification of MicroRNA Profiles in Fetal Spina Bifida: The Role in Pathomechanism and Diagnostic Significance

**DOI:** 10.3390/ijms25052896

**Published:** 2024-03-01

**Authors:** Angelika Buczyńska, Iwona Sidorkiewicz, Magdalena Niemira, Adam Jacek Krętowski, Piotr Węgrzyn, Przemysław Kosiński, Monika Zbucka-Krętowska

**Affiliations:** 1 Clinical Research Centre, Medical University of Bialystok, M. Sklodowskiej-Curie 24a, 15-276 Bialystok, Poland; magdalena.niemira@umb.edu.pl (M.N.); adamkretowski@wp.pl (A.J.K.); 2 Clinical Research Support Centre, Medical University of Bialystok, M. Sklodowskiej-Curie 24a, 15-276 Bialystok, Poland; iwona.sidorkiewicz@umb.edu.pl; 3 Department of Endocrinology, Diabetology and Internal Medicine, Medical University of Bialystok, M. Sklodowskiej-Curie 24a, 15-276 Bialystok, Poland; 4 Department of Obstetrics, Perinatology and Gynecology, Medical University of Warsaw, 63A Zwirki i Wigury, 02-091 Warsaw, Poland; piotr.wegrzyn@wum.edu.pl (P.W.); przemyslaw.kosinski@wum.edu.pl (P.K.); 5 Department of Gynecological Endocrinology and Adolescent Gynecology, Medical University of Bialystok, M. Sklodowskiej-Curie 24a, 15-276 Bialystok, Poland

**Keywords:** prenatal screening, miRNA, biomarker, spina bifida, miR-320e

## Abstract

Distinct miRNA expression patterns may reflect anomalies related to fetal congenital malformations such as spinal bifida (SB). The aim of this preliminary study was to determine the maternal miRNA expression profile of women carrying fetuses with SB. Therefore, six women carrying fetuses with SB and twenty women with euploid healthy fetuses were enrolled in this study. Using NanoString technology, we evaluated the expression level of 798 miRNAs in both plasma and amniotic fluid samples. A downregulation of miR-1253, miR-1290, miR-194-5p, miR-302d-3p, miR-3144-3p, miR-4536-5p, miR-548aa + miR-548t-3p, miR-548ar-5p, miR-548n, miR-590-5p, miR-612, miR-627-5p, miR-644a, and miR-122-5p, and an upregulation of miR-320e, let-7b-5p, miR-23a-3p, miR-873-3p, and miR-30d-5p were identified in maternal amniotic fluid samples in SB when compared to the control group. The target genes of these miRNAs play a predominant role in regulating the synthesis of several biological compounds related to signaling pathways such as those regulating the pluripotency of stem cells. Moreover, the maternal plasma expression of miR-320e was increased in pregnancies with SB, and this marker could serve as a valuable non-invasive screening tool. Our results highlight the SB-specific miRNA signature and the differentially expressed miRNAs that may be involved in SB pathogenesis. Our findings emphasize the role of miRNA as a predictive factor that could potentially be useful in prenatal genetic screening for SB.

## 1. Introduction

Myelomeningocele, also known as spina bifida (SB), is a non-lethal congenital birth defect of the central nervous system that occurs in 1–10 per 1000 live births worldwide [1]. SB is caused by the incorrect development of the spinal cord due to incomplete closure of the neural tube at 28 days of gestation, resulting in local nerve damage and the sequelae of non-communicating hydrocephalus [2,3]. The pathogenesis of SB is still unknown [4]. However, it has been proven that folic acid deficiency increases the risk of neural tube defects [5].

Most cases of SB are detected prenatally during the second-trimester scan performed between 18 and 22 weeks of gestation. In some selected cases, SB detection is possible even earlier, during the routine 11–13-week ultrasound scan (US) performed by an experienced sonographer [6]. In some countries, biochemical screening for SB based on maternal serum alpha-fetoprotein measurement is available. However, the detection rate is lower compared to ultrasound markers. Women diagnosed with congenital birth defects, including SB, are encouraged to undergo amniocentesis after the 15th week of gestation to exclude the most common chromosomal aberrations [7]. SB screening in most countries is currently based solely on US evaluation. Introducing a novel, non-invasive screening tool with high sensitivity and specificity could potentially identify a high-risk group and improve the accuracy of US [8]. miRNAs play critical regulatory roles in various physiological and pathological processes, including differentiation, proliferation, development, apoptosis, and oncogenesis. Therefore, they may serve as potential diagnostic biomarkers [9].

SB develops in the early stages of embryonic development, typically within the first weeks of pregnancy. However, before anatomical defects become visible through imaging methods such as ultrasound, molecular changes, such as alterations in miRNA expression, may occur at a stage that is not easily detectable with conventional diagnostic tools. In this study, we assessed miRNA in samples collected during a specific gestational period, considering that significant molecular events related to SB may take place earlier. Our goal was to understand whether miRNA profiles during this period could provide information about the risk of SB and whether they could serve as potential biomarkers for this condition [7,8,9]. Moreover, introducing miRNA expression patterns to the diagnostic protocol may improve SB detection and also aid personalized clinical management [10]. Since the pathogenesis of SB is still unknown, the comprehensive knowledge of SB development may introduce new treatment targets, improve surgical techniques, and enhance the prognosis for neonates [11,12,13]. Thus, the main objective of the current study was to evaluate the significance of miRNAs in the pathogenesis of SB. Amniotic fluid miRNA profiling was performed to uncover new molecular features of SB. Additionally, maternal circulating miRNAs were identified as novel non-invasive screening biomarkers for SB.

## 2. Results

### 2.1. Amniotic Fluid

**Figure 1:**
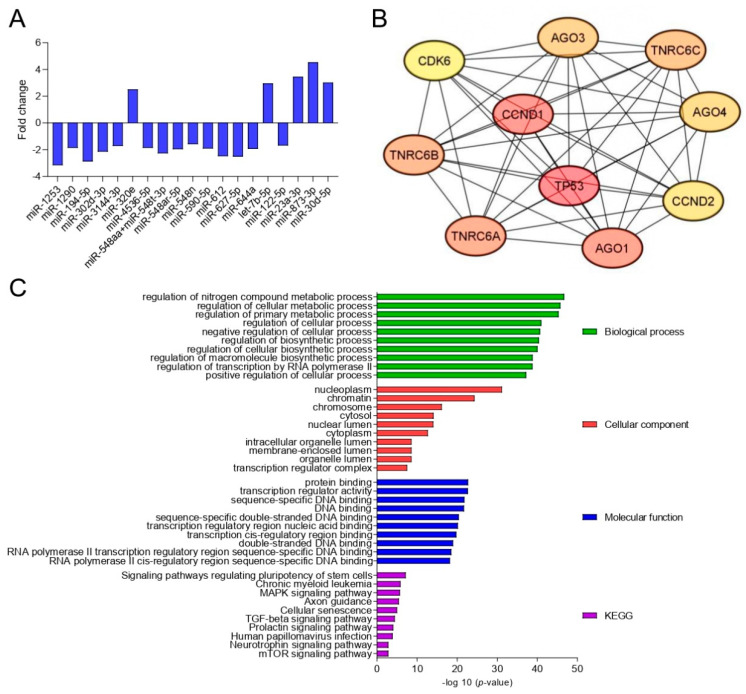
The amniotic fluid assessment in SB pathogenesis identification. (**A**) DE miRNAs in amniotic fluid with significantly different expressions between SB (n = 6) and control group (n = 20) (|FC| > 1.5; FDR < 0.05); (**B**) the network of the top 10 hub genes; (**C**) gene ontology (GO) enrichment analysis. Top 10 significantly enriched GO (−log10 (*p*-value)) terms of the target genes in the cellular components, molecular function, and biological processes. KEGG, Kyoto Encyclopedia of Genes and Genomes.

The amniotic fluid profiling of 798 miRNAs was performed using the NanoString Technology platform (Seattle, WA, USA), and 19 DE miRNAs were identified between the studied groups based on a threshold of an FDR < 0.05 and an |FC| ≥ 1.5 (Figure 1A).

We utilized the Cytoscape software version 3.8.2 to visualize the construction of the protein–protein interaction (PPI) network of target genes for the DE miRNAs obtained using MIENTURNET. To identify the most important genes in the PPI network, we applied the Maximal Clique Centrality (MCC) algorithm within the CytoHubba plugin. Consequently, we identified the top 10 hub genes, which included the TNRC gene family (trinucleotide-repeat-containing adaptor 6A (TNRC6A), trinucleotide-repeat-containing adaptor 6B (TNRC6B), trinucleotide-repeat-containing adaptor 6C (TNRC6C)), cyclin-dependent kinase 6 (CDK6), argonaute 3 (AGO3), argonaute 4 (AGO4), cyclin D2 (CCND2), argonaute 1 (AGO1), tumor protein 53 (PT53), and cyclin D1 (CCND1) (Figure 1B).

Gene ontology was used for identifying and visualizing the appropriate biological pathways and processes associated with the target genes (Figure 1C).

The analysis of the SB amniotic fluid network revealed a predominant role of the regulation of several biological compounds related to metabolic and synthesis processes, as well as incorrect functions of cellular components such as the neoplasm, chromatin, chromosome, cytosol, nuclear lumen, intracellular organelle lumen, and transcription regulator complex. With respect to molecular function terms, these genes were mainly enriched in transcriptional activator activity (RNA polymerase II core promoter proximal region sequence-specific binding), actin binding, sequence-specific DNA binding, and double-stranded DNA binding. The analysis of cellular components primarily identified those involved in the signaling pathways regulating the pluripotency of stem cells, mitogen-activated protein kinases (MAPK), transforming growth factor β (TGF-beta), prolactin, neurotrophin, and mammalian target of rapamycin (mTOR) signaling pathways. Additionally, incorrect neuronal development was indicated by axon-guidance-related processes.

**Figure 2:**
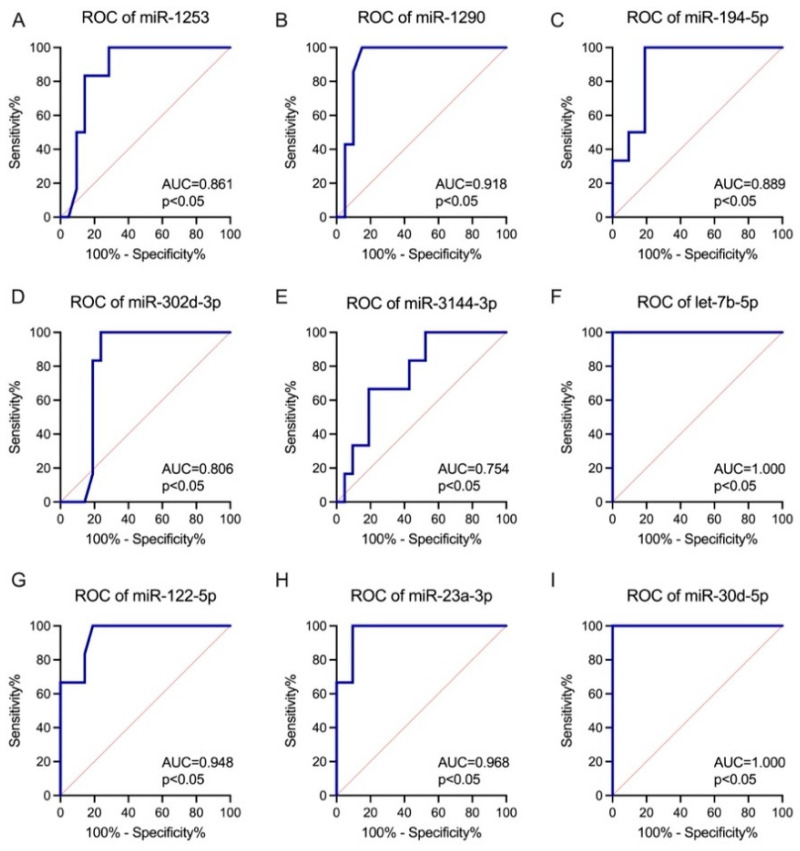
ROC analysis was constructed to evaluate the diagnostic values of the selected miRNAs as predictive biomarkers of SB (FC > 1.5; FDR < 0.05): (**A**) miR-1253; (**B**) miR-1290; (**C**) miR-194-5p; (**D**) miR-302d-3p; (**E**) miR-3144-3p; (**F**) let-7b-5p; (**G**) miR-122-5p; (**H**) miR-23a-3p; (**I**) miR-30d-5p.

**Figure 3:**
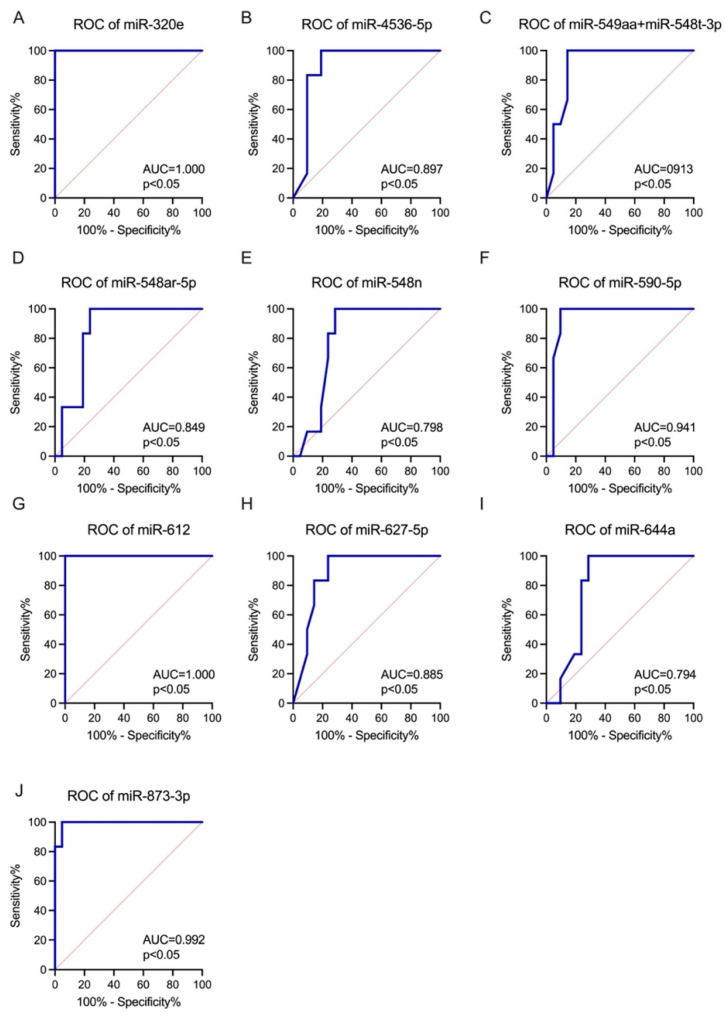
ROC analysis was constructed to evaluate the diagnostic values of the selected miRNAs in amniotic fluid as predictive biomarkers of SB (FC > 1.5; FDR < 0.05): (**A**) miR-320e; (**B**) miR-4536-5p; (**C**) miR-549aa + miR-548t-3p; (**D**) miR-548ar-5p; (**E**) miR-548n; (**F**) let-590-5p; (**G**) miR-612; (**H**) miR-627-5p; (**I**) miR-644a; (**J**) miR-873-3p.

Accordingly, the diagnostic value of the DE miRNAs as candidate SB biomarkers using the AUC was evaluated. All the identified DE miRNAs showed significantly higher AUCs compared with AUC = 0.50 (borderline of the diagnostic usefulness of the parameter). The highest AUCs, indicating possible clinical usefulness in SB diagnosis, were observed for let-7b-5p (AUC = 1.00), miR-30d-5p (AUC = 1.00), miR-320e (AUC = 1.00), miR-612 (AUC = 1.00), miR-23a-3p (AUC = 0.97), miR-122-5p (AUC = 0.95) and miR-590-5p (AUC = 0.94) (all, *p* < 0.05) (Figure 2 and Figure 3).

### 2.2. Maternal Plasma miRNA Profiling

**Figure 4:**
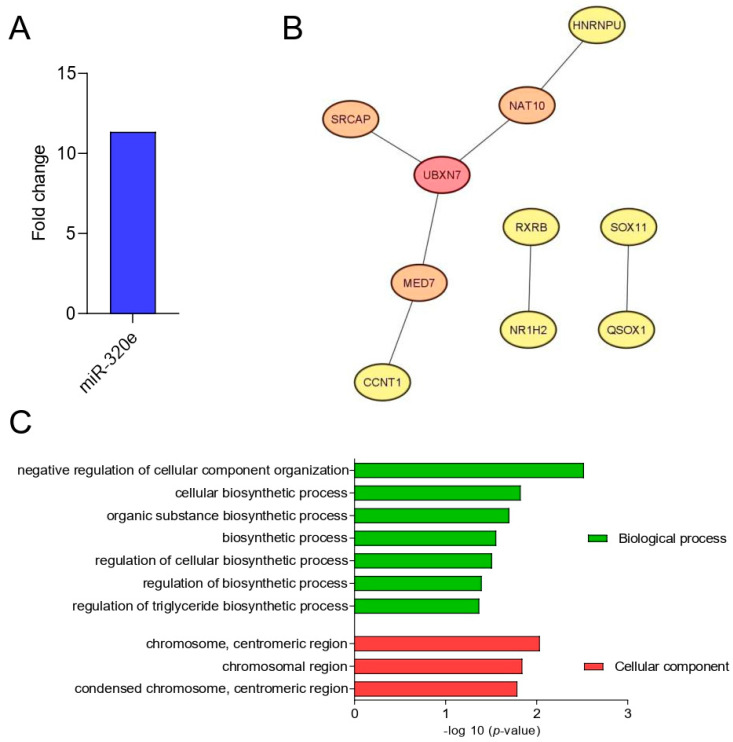
The plasma analysis among SB pathogenesis identifications. (**A**) The DE miRNAs in plasma with significantly different expressions between SB (n = 6) and control group (n = 20) (FC > 1.5; FDR < 0.05); (**B**) the networks of the top 10 hub genes targeted by miR-320e; (**C**) gene ontology (GO) enrichment analysis. Top significantly enriched GO (−log10 (*p*-value)) categories of the target genes in the cellular components, and biological processes.

To determine the SB-specific maternal miRNA expression pattern and to assess the potential utility of SB-specific miRNA as a screening tool, we profiled the plasma expression of 798 miRNAs using the NanoString Technology platform. The DE miRNA-320e was detected between the studied groups (*p* < 0.001) based on a threshold of an FDR < 0.05 and an |FC| ≥ 1.5 (Figure 4A).

We also predicted the DE miRNA-320e target genes using MIENTURNET. The results indicated 62 putative target genes for miRNA-320e, then the CytoHubba algorithm selected 10 hub genes. The STRING database was used to visualize the protein relationships (Figure 4B).

The UBX domain protein 7 (UBXN7) gene family linked to cyclin T1 (CCNT1), mediator complex subunit 7 (MED7), Snf2 related CREBBP activator protein (SRCAP), N-acetyltransferase 10 (NAT10), and heterogeneous nuclear ribonucleoprotein U (HNRNPU) with retinoid X receptor beta (RXRB), nuclear receptor subfamily 1 group H member 2 (NR1H2), SRY-box transcription factor 11 (SOX11), and quiescin sulfhydryl oxidase 1 (QSOX1) were the top 10 hub genes in the PPI network. The GO analysis of the miRNA network related to SB highlighted the predominant involvement of the negative regulation of cellular component organization and the cellular biosynthesis process among the most significantly associated biological processes. Additionally, the genes within the GO cellular component category were found to be connected to the centromeric region (Figure 4C).

**Figure 5:**
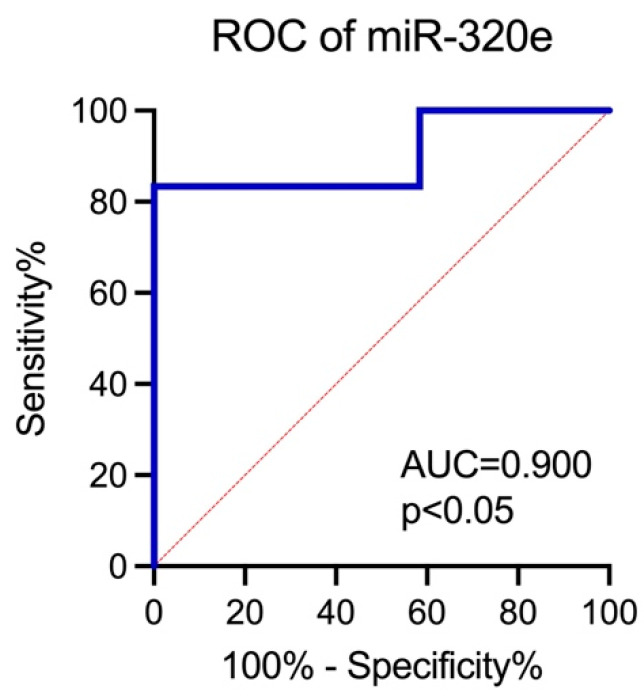
ROC analysis was conducted to evaluate the diagnostic value of miR-320e as diagnostic biomarker of SB vs. control.

Likewise, we assessed the diagnostic value of miR-320e as a potential biomarker for SB by evaluating the AUC. The analysis demonstrated that miR-320e exhibited the AUC value of 0.90, significantly increased when compared with AUC = 0.50, suggesting its potential clinical utility in SB screening (Figure 5).

### 2.3. Logistic Regression Model

A logistic regression model, a widely used supervised learning algorithm for binary classification tasks, was employed to examine the DE miRNAs and develop a screening signature for SB. Through the feature selection process, we identified two miRNAs that exhibited the most significant diagnostic values. Subsequently, a logistic regression model was constructed using these miRNAs. Notably, the combined use of miR-205-5p and miR-362-5p yielded a higher diagnostic value for SB (AUC = 0.94) compared to miR-320e (Table 1).

## 3. Discussion

The release of tissue-specific miRNAs in response to stress signals and their role as signaling molecules in health and disease highlights their potential as important tools for investigating deregulated pathways. Furthermore, miRNAs are involved in cellular communication and are likely to play important regulatory roles in maternal–fetal crosstalk [3,4,5,6]. Although the sample size in our study was limited and the low prevalence of SB among the general population should be considered, our findings provide valuable new insights into SB pathogenesis and the development of non-invasive biomarkers for prenatal screening. Therefore, identifying miRNAs associated with fetal SB provides significant contributions to understanding its pathogenesis.

Our study revealed the dysregulation of several miRNAs in amniotic fluid from fetuses with SB. Specifically, miR-1253, miR-1290, miR-194-5p, miR-302d-3p, miR-3144-3p, miR-4536-5p, miR-548aa + miR-548t-3p, miR-548ar-5p, miR-548n, miR-590-5p, miR-612, miR-627-5p, miR-644a, and miR-122-5p expression was downregulated and miR-320e, let-7b-5p, miR-23a-3p, miR-873-3p, miR-30d-5p expression was upregulated in SB when compared to the control group. There is little literature evidence to link the studied miRNAs to SB. Some of them have been connected to neoplastic disease development (miR-1253, miR-1290, miR-194-5p, miR-4536-5p, miR-548ar-5p, miR-590-5p, miR-627-5p, miR-320e, miR-23a-3p, miR-612, miR-23a-3p, miR-30d-5p, and miR-122-5p) [14,15,16,17,18,19,20,21,22,23,24]. The role of let-7b-5p in neuronal development and neuroinflammation has been demonstrated [25]. Li et al. showed a protective role of miR-23a-3p against neuronal injury by inhibiting neuron apoptosis and inflammatory response via reactivating the PTEN/AKT/mTOR signaling pathway [26]. It has been demonstrated that the upregulation of miR-23a-3p suppressed oxidative stress in ischemic brain injury using a mouse model [27]. Moreover, it has been shown that folic acid deficiency might upregulate miR-30d-5p to inhibit the expression of key genes in the endoplasmic reticulum stress pathway in colorectal cancer cells [28]. This suggests the possible involvement of miR-30d-5p in the cellular response to folic acid deficiency in SB pathogenesis.

Based on the role of miRNAs, it can be concluded that miRNA dysregulation results from the SB pathomechanism, rather than the dysregulated miRNAs actively participating in SB development. However, a better understanding of the mechanisms by which miRNAs are involved in SB may provide novel targets for innovative therapeutic strategies. To identify key DE miRNA target genes, a PPI network was constructed, revealing the TNRC gene family (*TNRC6A*, *TNRC6B*, and *TNRC6C*), *CDK6*, *AGO3*, *AGO4*, *CCND2*, *AGO1*, *PT53*, and *CCND1* as hub genes. Interestingly, *AGO* and the *TNRC6* family proteins have been shown to be the key players in the miRNA-induced silencing complex (miRISC), the machinery that mediates microRNA function in the cytoplasm [29]. One study established the importance of transcriptional regulation in neuronal differentiation with the central role of miRISC [30]. What should be noted is that the heterogeneity of the miRISC complex composition determines its dynamic function in regulating gene expression. Our results suggest the involvement of miRISC in modulating repressive activity in SB; however, the detailed impact of miRISC in SB pathogenesis is yet to be determined. Furthermore, *CCND1* and *CCND2* have been implicated in neurogenesis [31,32]. D-type cyclins (CCND1 and CCND2) are known to bind to CDK6 in response to mitogen exposure and activate the signal transduction cascade, resulting in cell division. The synchronized cell cycle progression allows for proper neural tube development [33]. The genetic background of SB is complex; however, our results suggest the involvement of both miRISC and cyclins. There is clearly a need for a detailed molecular study to understand their role in the SB pathogenesis.

Additionally, the GO analysis suggests that the signaling pathways regulating the pluripotency of stem cells, the MAPK signaling pathway, and axon guidance play important roles in the mechanism of T2DM development, which is consistent with the hub gene analysis. The study by Qin et al. demonstrated specific miRNA dysregulation (downregulation of miRNA-9 and miRNA-124a, and upregulation of miRNA-138 and miRNA-134) in amniotic fluid samples obtained from six pregnant rats with fetal SB compared to six control rats with healthy fetuses. The GO analysis revealed the involvement of these miRNAs in the MAPK signaling pathway, neurotrophin pathway, and mTOR signaling [34]. Li et al. demonstrated that neuroactive ligand–receptor interactions, the NF-kappa B signaling pathway, osteoclast differentiation, cytokine–cytokine receptor interactions, and cell adhesion molecules play crucial roles in the pathomechanism of SB [35].

Moreover, the miRNA profile in amniotic fluid may also represent an attractive diagnostic/prognostic biomarker. Let-7b-5p (AUC = 1.00), miR-30d-5p (AUC = 1.00), miR-320e (AUC = 1.00), miR-612 (AUC = 1.00), miR-23a-3p (AUC = 0.97), miR-122-5p (AUC = 0.95), and miR-590-5p (AUC = 0.94) (all, *p* < 0.05) have demonstrated high diagnostic value as SB biomarkers. Moreover, miR-320e has been shown to be significantly upregulated in the maternal plasma of patients with a fetus with SB compared to control maternal plasma. It can be hypothesized that the increased miR-320e expression in maternal plasma results from upregulation in the fetal compartment, as demonstrated in our study. The PPI analysis showed that *UBXN7*, *CCNT1*, *MED7*, *SRCAP*, *NAT10*, *HNRNPU*, *RXRB*, *NR1H2*, *SOX11*, and *QSOX1* were the top 10 genes regulated by the DE miRNA. UBXN7 is a cofactor of ubiquitin ligase complexes and mediates the reciprocal regulation of nuclear factor erythroid 2-related factor 2 (NRF2) and hypoxia-inducible factor (HIF) 1α proteins, directly involved in neurogenesis process, especially in the adaptation of neurons and glia cells to hypoxia and during neuronal differentiation [36,37,38]. The *CCNT1* gene has also been linked to the processes of neuron development by nucleic acid binding activity modulation [39]. *MED7* is a coactivator involved in the regulation of the transcription of nearly all RNA-polymerase-II-dependent genes, which was connected to folate-dependent cognitive impairment [40]. The *SRCAP* gene has been implicated in maintaining the self-renewal of embryonic stem cells, indicating its significant contribution to development. Moreover, *SRCAP*, a chromatin remodeler, promotes self-renewal in intestinal stem cells. On the other hand, *NAT10* has been shown to participate in various epigenetic processes such as histone acetylation and mRNA modifications [41]. The HNRNPU gene is implicated in encoding of the heterogeneous nuclear ribonucleoprotein U, a protein that plays essential roles in RNA splicing and chromatin organization. When its function is impaired, it results in the rapid cell death of both post-mitotic neurons and neural progenitors [42]. The RXRB gene encodes a protein that belongs to the retinoid X receptor (RXR) family of nuclear receptors. These receptors play crucial roles in mediating the effects of retinoic acid, especially in the induction of neurogenesis by activating both retinoic acid receptors (RARs) and the peroxisome proliferator-activated receptor β/δ (PPARβ/δ) [43,44]. NR1H2, a nuclear receptor, plays a key role in the regulation of cholesterol uptake through myosin regulatory light chain interacting protein (MYLIP)-dependent ubiquitination, and the regulation of the lipid-induced stress response and inflammation, particularly involved in disturbed neurogenesis and spinal cord injury [45]. The study performed by Wang et al. showed that *SOX11* is essential for both embryonic and adult neurogenesis, by transient proliferation deficits in neural progenitor cell proliferation [46,47]. Moreover, *QSOX1* has been associated with neural tube defect development via its interaction with valproic acid and folic acid [12]. The potentially disturbed function of genes indicated by DE miRNAs suggests involvement in the processes of neurogenesis, responses to oxidative stress, lipid metabolism, and the transport and distribution of folic acid, which confirms the complex pathogenesis of developmental defects in SB. Thus, more extensive research in this field is still needed.

Furthermore, the GO analysis demonstrated a predominant role of target genes in the negative regulation of cellular component organization and biosynthetic processes, indicating their role in cellular response and metabolism in SB.

Plasma miRNA profiling may also be a useful tool for the non-invasive prenatal screening of SB. Thus, we evaluated the diagnostic value of miR-320e. Since only one miRNA was differentially expressed in our study group when assessed in maternal plasma, all miRNAs in the NanoString panel were screened for their significance when combined into a diagnostic panel. The feature selection chose two miRNAs that did not show differential expression—miR-205-5p + miR-362-5p—and the logistic regression model for the comparison of SB vs. control showed their higher diagnostic value (AUC = 0.94) compared to the AUC found for miR-320e (AUC = 0.90) when assessed in maternal plasma.

miR-205 has garnered significant attention due to its involvement in both normal developmental processes and cancer. During early embryogenesis, miR-205 is expressed in trophoblasts and contributes to placental development by inhibiting the expression of mediator of RNA polymerase II transcription subunit 1 (MED1) [48]. As embryonic development progresses, miR-205 also plays a role in regulating extraembryonic endoderm differentiation and spermatogenesis by targeting genes associated with cell migration and adhesion [49]. Notably, miR-362 is associated with specific medical conditions such as polymyositis and dermatomyositis. Functional studies have further revealed that miR-362 exerts inhibitory effects on the proliferation and migration of vascular smooth muscle cells [50]. These findings raise the possibility of miR-362’s involvement in SB development.

In the context of embryonic development, both miR-205 and miR-362 present intriguing roles that warrant further investigation, particularly regarding their potential participation in processes such as neural tube formation. Such processes are of particular significance in the context of SB development. However, it is crucial to emphasize that our analyses should be considered preliminary, and their validation through larger-scale studies is imperative to confirm their validity [51]. The results of our study are in line with the current knowledge about SB pathogenesis. The expression pattern of miRNAs in SB pregnancies may impact further improvements in prenatal screening by uncovering disturbed molecular pathways.

### Study Limitations

While our study demonstrates promising findings regarding the potential of miRNA markers as non-invasive screening tools for SB, it is essential to acknowledge that screening strategies may not directly translate into preventive measures. Despite the high screening accuracy observed for miR-320e in maternal plasma, it is imperative to recognize the limitations and complexities associated with implementing screening programs in clinical practice. Firstly, the transition from screening to prevention requires a comprehensive understanding of the underlying biological mechanisms and modifiable risk factors associated with SB. While our study contributes to elucidating the SB-specific miRNA signature, further research is warranted to investigate the causal relationships between miRNA dysregulation and SB pathogenesis. Additionally, identifying environmental and genetic factors that interact with miRNA expression could enhance risk stratification and inform targeted preventive interventions. Moreover, the effectiveness of screening programs depends not only on diagnostic accuracy but also on factors such as accessibility, acceptability, and cost-effectiveness. Implementing widespread screening for SB based on miRNA markers would necessitate robust evidence supporting their clinical utility, as well as considerations of resource allocation and ethical implications.

Our study was also not without limitations, the main among them being a small sample size, which resulted from the low incidence of SB in the general population. However, our sample size does not differ significantly from that of other similar studies [51,52]. Therefore, the results of our study should be considered preliminary, and they require blind validation in a large patient cohort to confirm the significance of differential miRNA expression in SB screening. While we have collected and analyzed data from samples obtained during amniocentesis, it is crucial to recognize that this procedure is typically performed during the second trimester of pregnancy, typically between the 15th and 18th weeks of gestation. This timing places the collection of amniotic fluid samples at a stage of pregnancy significantly later than when the primary events leading to SB take place. The primary events, including neural tube closure, occur during the initial stages of pregnancy, often before many women are even aware of their pregnancy. Therefore, the miRNA profiles or other biomarkers that we are assessing in the amniotic fluid represent a later developmental stage compared to the critical period when SB initiation and progression primarily occur. Consequently, when interpreting our results, it is imperative to consider this temporal gap between the events of interest and the timing of sample collection. The miRNA profiles observed in the amniotic fluid may reflect a downstream consequence of earlier developmental processes and may not fully capture the initial molecular events associated with SB etiology.

## 4. Materials and Methods

### 4.1. Material Collection

An adequate sample size to detect a difference was demonstrated using power analysis. Considering a 95% confidence level, 5% margin of error, and SB incidence, a minimum of 6 samples were required for this study. Therefore, the study group consisted of 6 pregnant women at the Department of Obstetrics, Perinatology and Gynecology, Medical University of Warsaw, Poland, in whom SB was detected prenatally. All cases of lesions were of the open spina bifida type at levels between L1 and S1. The control group comprised 20 women who underwent routine amniocentesis at the Department of Reproduction and Gynecological Endocrinology of the Medical University of Bialystok, Poland. All 26 patients underwent amniocentesis during the gestational period between the 15th and 18th weeks, and all subsequent samples, consisting of 26 plasma and 26 amniotic fluid samples, were included in the analysis.

The exclusion criteria were chronic or acute diseases, hormonal or/and anti-inflammatory treatment. All participants were informed about potential risks prior to the procedure and received relevant information regarding the study. The study participants were matched according to age, ethnicity, socioeconomic status, the course of pregnancy, body mass index (BMI), and the number of pregnancies with marked episodes of pregnancy pathology. All participants in the study were meticulously selected according to specific criteria, including an age range of 20 to 40 years, self-identification as belonging to the Caucasian ethnic group with Eastern European heritage, absence of comorbidities, and no history of chronic medication usage. Statistical analysis demonstrated no significant differences between the studied groups across all criteria (*p* > 0.05). Both amniotic fluid (5.0 mL) and venous blood (5.5 mL) samples were obtained from participants on the same day. Venous blood was centrifuged, and, subsequently, both plasma and amniotic fluid samples were transferred into DNase- and RNase-free tubes (Eppendorf, Hamburg, Germany), and stored at −80 °C until assayed. The methods were carried out in accordance with approved guidelines and conducted in accordance with the ethical standards of the institutional research committee, with the Helsinki Declaration, and were approved by the local ethics committee of the Medical University of Bialystok, Poland (approval number: APK.002.76.2021).

### 4.2. nCounter miRNA Expression Assay

The nCounter miRNA Expression pre-designed panel simultaneously detects 798 human miRNAs, including five mRNA reference controls (B2M, GAPDH, RPL19, ACTB, RPLP0). Positive and negative proprietary spike-in controls (probes that recognize exogenous miRNA targets to monitor upstream RNA isolation/purification; osa-miR442, osa-miR414, cel-miR-254, and cel-miR-248), hybridization controls and ligation-specific controls were also included to determine sample integrity, quality, and background. The NanoString platform provided several key advantages, including sensitivity and robustness for analyses of plasma samples. This assay involves sample processing where unique oligonucleotide tags are annealed and subsequently ligated with miRNAs of interest via a target specific bridge oligo. Sequence specificity between each miRNA and its appropriate tag is ensured by careful, stepwise control of annealing and ligation temperatures. NanoString counts single molecules with no amplification step. The nCounter miRNA Expression Assay requires purified total RNA as input material.

miRNA was isolated from 200 µL of plasma and amniotic fluid samples using the miRNeasy Serum/Plasma Advanced Kit (Qiagen, Hilden, Germany), following the manufacturer’s instructions. miRNA was eluted in a final volume of 15 μL.

The RNA concentration was measured using Qubit (Invitrogen, Carlsbad, CA, USA) and 100 ng total RNA was used as input material. Next, the samples were prepared for nCounter miRNA expression profiling as per the manufacturer’s recommendations (NanoString Technologies, Seattle, WA, USA). Briefly, sample preparation involved a multiplexed annealing of the specific tags to their target miRNA, a ligation reaction, an enzymatic purification to remove unligated tags, followed by an overnight hybridization (65 °C) with nCounter Reporter and Capture probes. Subsequently, the samples were placed into the nCounter Prep Station for automated sample purification and reporter capture. Each sample was scanned (550 fields of view) on the nCounter Digital Analyzer for data collection. The NanoString data were deposited in the Gene Expression Omnibus (GEO) database (Accession Number: GSE242364).

### 4.3. Data Analysis

The nSolver 4.0 Analysis software (NanoString) was utilized for data analysis, which included a normalization step using the ligation method. The *p*-values were adjusted using the false discovery rate (FDR) correction for multiple comparisons, which was limited to 0.05 for both plasma and amniotic fluid samples. A threshold value for significance of fold change |FC| of ≥1.5 was applied to define differentially expressed (DE) miRNAs. Statistical analyses were conducted using GraphPad PRISM (v.9.1.1; GraphPad Software, San Diego, CA, USA). To assess the screening utility, the receiver operating characteristic (ROC) curves were analyzed and the area under the ROC curves (AUC) was calculated.

### 4.4. miRNA Target Prediction and Functional Annotation

To examine the functions of the identified miRNAs, miRNA target prediction was performed using MicroRNA Enrichment Turned Network (MIENTURNET, Rome, Italy; http://userver.bio.uniroma1.it/apps/mienturnet/, accessed on 15 February 2023), which utilizes TargetScan and miRTarBase databases. To identify highly connected hub genes in the protein–protein interaction (PPI), the Search Tool for Retrieval of Interacting Genes/Proteins (STRING) was used (accessed on 15 February 2023), with the interaction score > 0.4. The CytoHubba plugin based on Cytoscape version 3.8.2 (http://cytoscape.org, accessed on 16 February 2023) was applied. To examine the functions of the identified target genes, gene ontology (GO) analysis and functional annotation clustering were carried out using the following online databases: Gene Ontology enrichment analysis and visualization tool (GOrilla; http://cbl-gorilla.cs.technion.ac.il/, accessed on 16 February 2023), DAVID (Gene Ontology and KEGG Enrichment Analysis; https://david.ncifcrf.gov/, accessed on 16 February 2023), g: Profiler (https://biit.cs.ut.ee/gprofiler/gos accessed on 16 February 2023), and Metascape (https://metascape.org, accessed on 16 February 2023). GO analysis allows for associating a given gene list with specific functional annotations, which are further divided into functional clusters listed according to their enrichment *p*-value.

### 4.5. Modeling

To construct a model that combines miRNAs for the diagnosis of fetal SB, we used Weka software (v.3.8.6; Hamilton, New Zealand). Attribute selection was performed using the Info Gain evaluator and Ranker search to select miRNAs with the highest ranks for further modeling. Logistic regression, naive Bayes, and J48 tree-based algorithms were calculated for each combination, and confusion matrices were prepared for each model to evaluate its performance. To control for errors, we used the “Leave One Out Cross-Validation” method, as the sample size was limited. The performance of the multi-miRNA classifiers was evaluated using the classification precision and the AUC.

## 5. Conclusions

Our analysis demonstrates the usefulness of evaluating miRNA expression patterns in the prenatal diagnosis of spinal bifida (SB). Additionally, a screening panel comprising miR-205-5p + miR-362-5p and miR-320e may be introduced as a potential tool for the maternal serum prenatal screening of SB. Furthermore, characterizing the regulatory network of DE miRNAs and target genes in amniotic fluid is important for investigating the pathways that contribute to a better understanding of SB pathogenesis. Nonetheless, further studies are required to understand the role of dysregulated miRNAs in the molecular mechanisms underlying the occurrence of SB-related comorbidities.

## Figures and Tables

**Table 1:** Maternal circulating miRNA panel for SB screening.

PANEL	TP Rate	FP Rate	Precision	AUC	Intercept	Coefficients
**x1 = miR-205-5p** **x2 = miR-362-5p**	0.833	0.168	0.844	0.944	13.9851	a1 = −19.2974 a2 = 0.2402

TP Rate—true positive rate; FP Rate—false positive rate; AUC—area under the receiver operating characteristic curve.

## Data Availability

The datasets used and/or analysed during the current study available from the corresponding author on reasonable request.
